# Rare Variant of Adult Rhabdomyosarcoma Presenting as a Palatal Swelling

**DOI:** 10.12669/pjms.37.3.3305

**Published:** 2021

**Authors:** Hafiz Aamer Iqbal, Rabia Anjum, Nadia Naseem

**Affiliations:** 1 Dr. Hafiz Aamer Iqbal, BDS; FCPS (Oral and Maxillofacial Surgery) Department of Oral & Maxillofacial, Surgery Services Hospital, Lahore, Pakistan; 2 Dr. Rabia Anjum, BDS; MPhil (Oral Pathology) Assistant Professor, Department of Oral Pathology, University of Health Sciences, Lahore, Pakistan; 3 Prof. Nadia Naseem, MBBS; PhD (Histopathology) Head, Department of Morbid Anatomy and Histopathology, University of Health Sciences, Lahore, Pakistan

**Keywords:** Immunohistochemistry, Malignant spindle cell neoplasm, Oral Cavity, Rhabdomyosarcoma

## Abstract

A 26-year-old male was referred to the Department of Oral and Maxillofacial Surgery of a tertiary care hospital in Lahore with chief complaint of painless swelling on the right palate of 40 days duration. Clinical differential diagnosis included squamous cell carcinoma, Ewing sarcoma, fibrosarcoma, neuroblastoma and rhabdomyosarcoma. Computed tomography scan revealed hypodense mass with necrotic changes. Histological examination of the excised tumor revealed malignant neoplasm arranged in fascicles and bundles comprising of spindle cells with pleomorphic, hyperchromatic nuclei and increased atypical mitosis. Immunohistochemical analysis showed negative staining with Cytokeratin, S100, CD34, Stat6, h-Caldesmon and EMA while the tumour cells were positive for desmin, myogenin, smooth muscle actin, CD-99 and MyoD1 thus confirming the diagnosis of spindle cell rhabdomyosarcoma.

## INTRODUCTION

Rhabdomyosarcoma (RMS) is malignant soft tissue neoplasm comprising of cells of primitive mesenchymal origin with a strong tendency of myogenesis. The tumor arises within a skeletal muscle but can also be found in areas lacking muscle tissue, such as the salivary glands, base of the skull, biliary tree, and genitourinary tract.^1^ RMS is common in childhood however occurs rarely in adults representing 3% of all adult sarcomas.^2^ Among Head & Neck RMS, oral cavity is one of the rare sites of origin. This tumor shows aggressive clinical course in the adult population with rapid spread to surrounding vital structures. Histologic variants of RMS include embryonal, alveolar, pleomorphic and spindle cell types according to latest WHO classification.^3^ Spindle cell variant is rare and is reported to have better prognosis than other variants. The histological variants of rhabdomyosarcoma may mimic other sarcomas on plain histology therefore, to confirm the diagnosis immunohistochemistry is mandatory. We present a case of oral rhabdomyosarcoma arising on the right side of palate in a 26-year-old male.

## CASE REPORT

**Fig.1:**
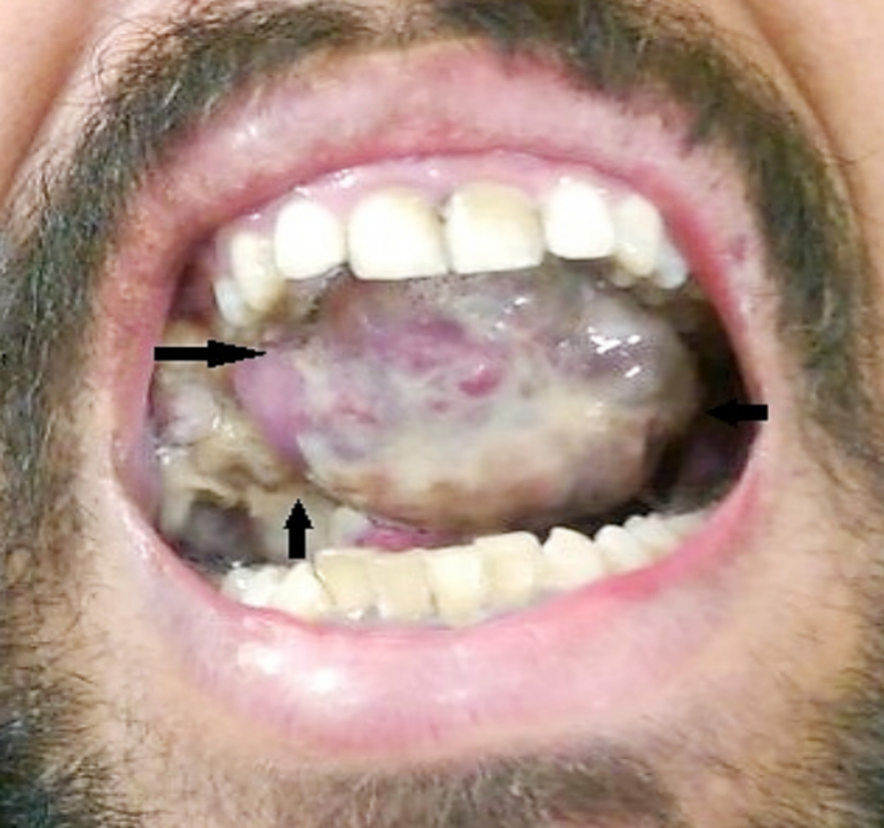
Painless swelling in right side of the palate. Arrows indicating clinical boundaries of swelling.

**Fig.2:**
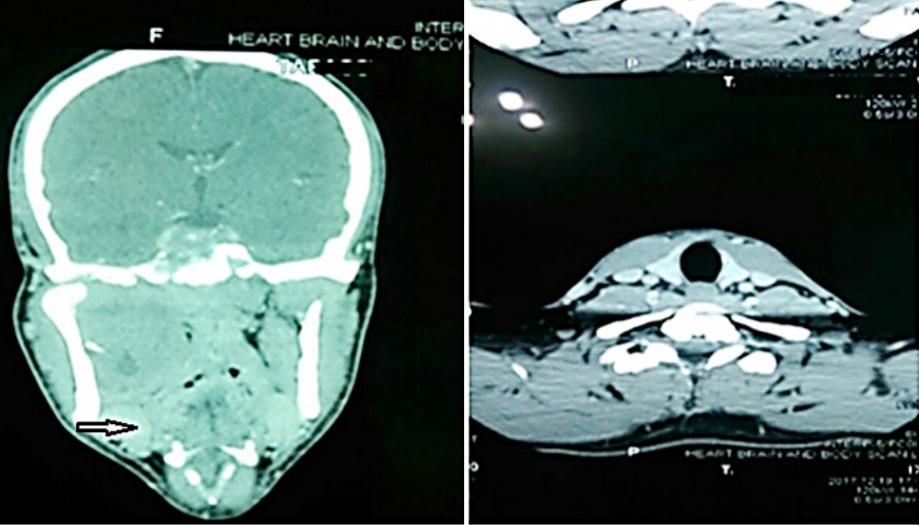
CT scan of face and neck is showing hypodense mass (→). Erosive changes are evident in lateral wall of the right orbit (B).

A 26-year-old male reported to the Department of Oral and Maxillofacial Surgery of a tertiary care hospital in Lahore Pakistan with a chief complaint of painless swelling on the right side of palate of 40 days duration (Fig.1). On clinical evaluation, a malignant tumor was suspected and differential diagnosis of squamous cell carcinoma, Ewing sarcoma, fibrosarcoma, neuroblastoma and rhabdomyosarcoma were considered. Computed tomography scan of the face and neck revealed 60mm×60mm×51mm hypodense mass with necrotic changes extending from the base of skull and infratemporal fossa involving right pterygoid muscles and parapharyngeal space with erosion of right posterolateral maxillary sinus. Erosive changes were also evident in lateral wall of the right orbit, skull base, body of sphenoid, upper basis sphenoid and right pterygoid plate. Mild soft tissue thickening in the region of right cavernous sinus was observed with moderate right ostomastoiditis. Right carotid sheath showed mild posterior displacement. However, no proptosis or vascular encasement was seen. A differential diagnosis of Ewing Sarcoma, Rhabdomyosarcoma, Osteosarcoma and Fibrosarcoma was formulated (Fig.2).

**Fig.3:**
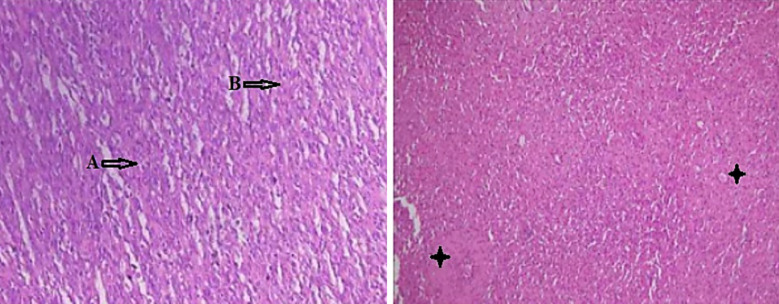
Photomicrograph shows malignant neoplasm arranged in fascicles and bundles comprising of spindle cells with pleomorphism, hyperchromatic nuclei and increased mitosis (A&B). Stars (♦) indicate the areas of necrosis and intervening hemangiopericytoma can also be seen. (H&E, 40X)

Incisional biopsy was performed, and tissue was submitted for histopathological examination. Grossly, specimen consisted of multiple soft whitish tissue fragments collectively measuring 1.5cm×1.0cm×0.8cm. Microscopic examination revealed moderately cellular neoplasm composed of sheets of small, stellate cells with scant deeply eosinophilic cytoplasm and eccentric oval nuclei with light chromatin pattern and inconspicuous nucleoli. There was increased mitotic activity with numerous atypical mitoses seen. Immunohistochemical analysis showed negative staining for Cytokeratin, CAM5.2, ALK and SMA while Desmin, S100 and Myogenin showed positivity. Based on clinical, radiological & histological findings the tumor was suspected as rhabdomyosarcoma. However, request was made for the excisional biopsy of the tumour to confirm the diagnosis and to rule out other similar lesions. Excisional biopsy contained multiple pieces of tissue fragments of light brown color, measuring 8cm × 7.0cm × 4.0cm. Histological examination of the submitted sections revealed malignant neoplasm arranged in fascicles and bundles comprising of spindle cells with pleomorphic, hyperchromatic nuclei and increased mitosis. Foci of necrosis and intervening hemangiopericytoma like vascular pattern was also present. The spindle cell morphology in the tumor created diagnostic challenges, thereby an immunohistochemical staining using a panel of markers for screening was used. Histopathological differential diagnosis of malignant spindle cell tumour was made (Fig.3) and IHC was advised to confirm the diagnosis.

**Fig.4:**
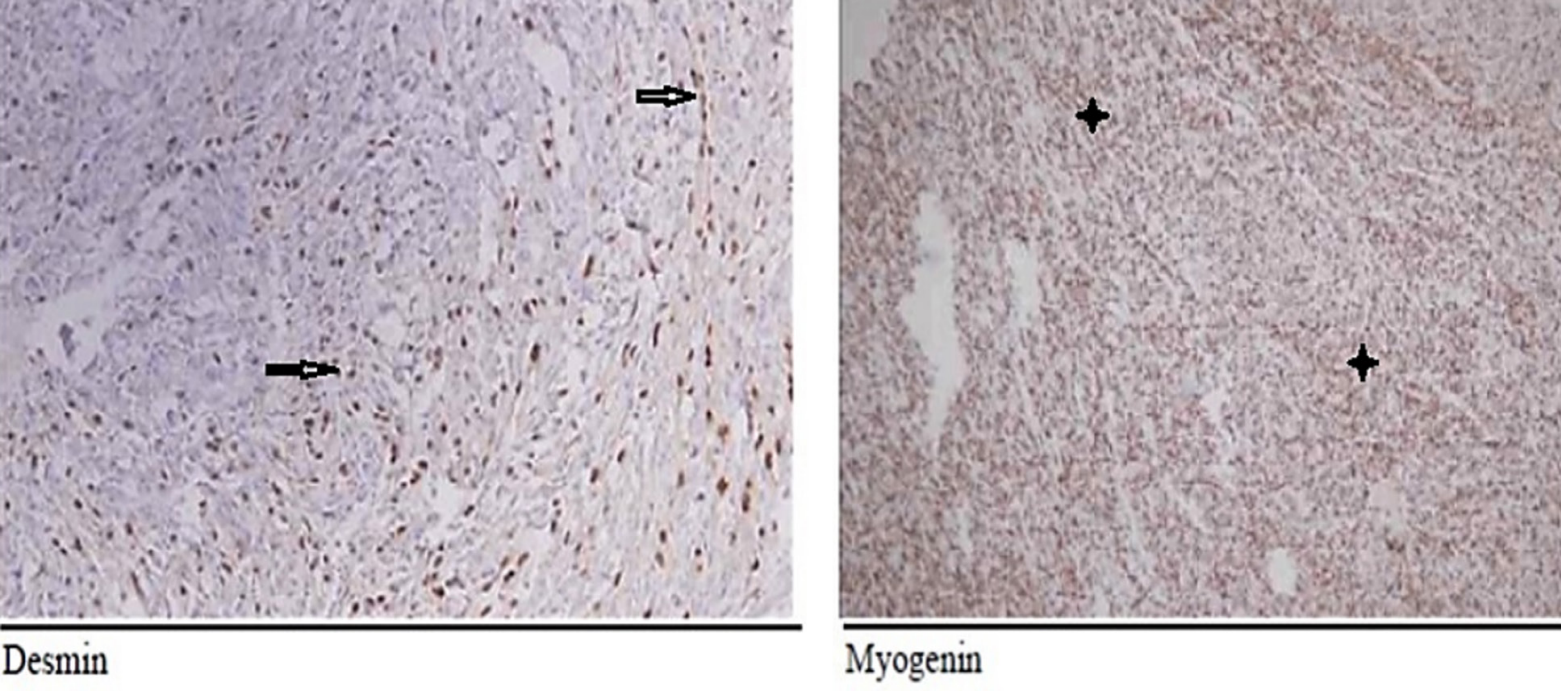
Immunohistochemical analysis shows positive staining for Desmin (→) & Myogenin(♦).

The tumour cells were found to be positive for desmin, myogenin, SMA, CD-99, MyoD1 while negative for cytokeratins, S100, CD34, Stat6, h-Caldesmon and EMA (Fig.4). A final diagnosis of spindle cell rhabdomyosarcoma was made considering the history, clinical, radiographic and histopathological findings. The patient was kept on regular follow up and remained tumor free at one-year follow-up.

## DISCUSSION

In the oral cavity only 1-2% of the malignant tumors are sarcomas, of which rhabdomyosarcoma is rare. Literature shows male predominance with a male: female ratio of 2.1:1.^4^ Oral RMS has been reported in adults with median age of 21 years.^5^ Similar findings of age and gender were observed in the present case. Oral cavity as a primary site of RMS is rare with few studies reporting involvement of soft palate, tongue, buccal mucosa, maxillary sinus, and posterior region of mandible.^6^ Chen et al (2015) reported seven cases, out of which only one had occurred on the palate.^5^ The reported cases are generally aggressive lesions extending to the adjacent alveolus, soft palate, oropharynx and nasopharyngeal areas. The study by Parviz D (2014) reported infiltrative soft tissue mass causing facial asymmetry, marked expansion into adjacent structures, and lingual cortical plate perforation on Computed Tomography (CT).^7^ Similar findings on CT were observed in current case. Generally, oral rhabdomyosarcoma is seen as advanced lesions at the early stage because it rapidly enlarges as a painless mass infiltrating locally. Pain, ankyloglossia, paresthesia, and trismus can also be the presenting symptoms with these lesions.^7^ The chief complaint in present case was painless swelling on the right side of the palate.

In oral cavity the initial clinical presentation of rapidly growing spindle cell lesions leads to suspicion of sarcomatoid carcinoma^8^ followed by spindle cell malignancies that may include leiomyosarcoma, malignant peripheral nerve sheath tumor with heterologous rhabdomyoblastic differentiation, and fibrosarcoma. The presence of fascicles of spindle cells with pleomorphism and hyperchromatic nuclei in the present case pointed towards diagnosis of spindle cell variant of rhabdomyosarcoma and malignant spindle cells, fibrosarcoma and leiomyosarcoma, however, there was absence of herring bone and storiform patterns. In all such cases, immunohistochemistry is required to confirm the diagnosis. Recommended markers to determine the skeletal muscle differentiation are desmin and MyoD. The MyoD family contains four different proteins myogenin, MyoD1, myf-5, and MRF-4. Among these Myogenin and MyoD1 are highly sensitive and specific for differentiation of skeletal muscle tissue.^9^ Positive reaction for desmin indicates myogenic differentiation but has low specificity for differentiating between skeletal and smooth muscle cells. The diagnosis of rhabdomyosarcoma can be made only if at least one of these proteins show positivity. In current case, desmin, myogenin, SMA (Smooth muscle Actin) MyoD1 and CD99, all were positive.

RMS rarely occurs in the oral cavity of adults with few cases arising in the hard palate. The histopathological subtypes most commonly found are embryonal, alveolar, and pleomorphic. After revised WHO classification, Spindle cell variant of RMS has been reported in oral cavity in approximately 5%-13% of the cases.^10^ This rare variant should always be considered in case of an aggressive palatal swelling in adults.

## CONCLUSION

A case of rhabdomyosarcoma is reported in a 26-year-old male with a palatal swelling. The tumor is rare and has an aggressive nature with a high recurrence potential therefore accurate and timely diagnosis becomes crucial. Immunohistochemical markers should always be employed in case spindle cell neoplasms are detected in the palate of adults to rule out spindle cell variant of oral RMS.

### Authors Contribution:

**HAI:** Collection of Data, critical revision of the manuscript for intellectual content.

**RA:** Design and drafting of manuscript

**NN:** Design and critical revision of the manuscript for intellectual content.

All authors are responsible for responsible and accountable for the accuracy or integrity of the work.
